# (Hu)man-Like Robots: The Impact of Anthropomorphism and Language on Perceived Robot Gender

**DOI:** 10.1007/s12369-023-00975-5

**Published:** 2023-03-21

**Authors:** Eileen Roesler, Maris Heuring, Linda Onnasch

**Affiliations:** Department of Psychology and Ergonomics, Technische Universität Berlin, Marchstr. 12, 10587 Berlin, Germany

**Keywords:** Anthropomorphism, Appearance, Gender associations, Language, Male-robot bias

## Abstract

Implementing anthropomorphic features to robots is a frequently used approach to create positive perceptions in human–robot interaction. However, anthropomorphism does not always lead to positive consequences and might trigger a more gendered perception of robots. More precisely, anthropomorphic features of robots seem to evoke a male-robot bias. Yet, it is unclear if this bias is induced via a male appearance of higher anthropomorphic robots, a general male-technology bias, or even due to language aspects. As the word robot is differently grammatically gendered in different languages, this might be associated with the representation of robot gender. To target these open questions, we investigated how the degree of anthropomorphism and the way the word robot is gendered in different languages, as well as within one language influence the perceived gender of the robot. We therefore conducted two online-studies in which participants were presented with pictures of differently anthropomorphic robots. The first study investigated two different samples from which one was conducted in German, as grammatically-gendered language, and one in English as natural gender language. We did not find significant differences between both languages. Robots with a higher degree of anthropomorphism were perceived as significantly more male than neutral or female. The second study investigated the effect of grammatically-gendered descriptions (feminine, masculine, neuter) on the perception of robots. This study revealed that masculine grammatical gender tends to reinforce a male ascription of gender-neutral robots. The results suggest that the male-robot bias found in previous studies seems to be associated with appearance of most anthropomorphic robots, and the grammatical gender the robot is referenced by.

## Introduction

Robots are increasingly entering our private and work environments, resuming diverse tasks from industrial manufacturing to social companionship. Due to this plethora of tasks and applications, robots with a wide variety of morphologies have been developed and implemented [1]. One trend with regard to robot design that is gaining ground in all domains, however, is the use of anthropomorphic features [2]. The basis for the effectiveness of anthropomorphism by design [3] is the human tendency to anthropomorphize. This tendency is a stable individual difference, which can predict how humans perceive agents [4]. Using this tendency, the design approach aims to trigger the perception of human-like characteristics in robots by implementing according design features like facial features, natural communication, or movements [1]. It is assumed that such features enable a more intuitive interaction by offering cues referring to social scripts from human-human interaction [5, 6]. However, this transfer of scripts might not only be associated with an intuitive interaction, but also comprise other social categories that might be (unintentionally) transferred to human-robot interaction (HRI), too.

In this context, one of the most common ways to categorize others in social interactions, is binary gender (female vs. male) [7]. Social categories are often treated as if they were natural categories, and the category most strongly associated with being a natural kind is gender, followed by race and ethnicity [8]. This categorization goes along with the activation of gender-stereotypical expectations about appearance, traits, behavior, or interests [9] as well as social roles in regard to e.g., instrumental competence or emotional sensitivity [10].

In HRI, some robot designs aim to take advantage of this linkage between perceived gender and assumed characteristics of social roles [11]. Some research already illustrated that robots are evaluated more positively and accepted more when their gender in regard to voice and name matched to the gender-occupational role of the job they should perform (healthcare vs. security) [12]. In line with this, the perceived suitability of robots for different tasks depends on their ascribed gender [13]: Stereotypically male tasks like transporting goods or repairing technical devices were perceived as more suitable for a male robot, whereas a female robot was perceived more suitable for stereotypically female tasks like care services or household maintenance. As the aforementioned studies exemplify, gendering robots is often justified by assumed beneficial effects on the expectations on part of the human, for example that female robots are associated with higher emotional intelligence [14]. However, also a mismatch between robot gender and gender stereotype of the task offers the opportunity to facilitate HRI [15]. More precisely, the willingness to engage in a learning process can be higher if the robot gender is the opposite of the stereotype of the learning task (e.g., literature as stereotypically female and mathematics as a stereotypically male task.)

Whereas a mismatch of robot gender and gender stereotype of the task might be a useful approach to counter gender bias in HRI, other research questions whether gendering robots, hence transferring stereotypes to them, is really necessary at all. Especially, as gendered robots might further reinforce existing occupational stereotypes, gender-neutral robots seem to offer the opportunity to counteract this effect. This assumed advantage is supported by a current study [16], revealing that trust in occupational competence was not significantly different for male, female, or gender-neutral robots. Thus, gender-neutral robots seem to show no disadvantages in work-related perception while reducing corresponding stereotypes.

However, even though non-gendering might be the desirable approach in robot design, gender associations might still come as unintended side effects [17]. Even if robots are not explicitly assigned a gender by appearance via hair length, lip color, and proportions or context via names and pronouns, existing occupational stereotypes could still lead to the categorization of gender-neutral designed robots as male or female. For example, a current experiment [18] investigated how robots without obvious gender cues are categorized in terms of gender in different application domains. Findings revealed that the prevalent association of robots was a gender-neutral or functional one. In addition, the results showed that if robots were gendered, they were significantly more categorized as male than female in all domains (i.e., social, service, industrial). The results therefore seem to indicate rather a cross-domain male-robot bias than the transfer of domain-specific stereotypes. This is in line with the general strong link of technology with masculinity [19]. On the one hand, men are still dominantly developing and implementing technologies, or working more closely together with technologies (e.g., in blue collar work) [20]. On the other hand, the physical strength and/or computational capabilities of machines are associated with masculinity [21]. It therefore seems reasonable that also a general male robot-bias might exist.

In order to further support the assumption of a male-robot bias by empirical evidence, two central methodological criticisms of the mentioned study [18] however have to be considered: First, even though the robots used did not represent obvious gender cues in appearance [18], especially the more anthropomorphic robots might have still been perceived as male gendered because of their body proportions [22]. Second, the gender perception of the robots might be influenced by the native language of the participants. As the study was conducted with German participants and German is a grammatically gendered language, this might have affected results.

The language people speak influences how they think about the world they live in. Even though in the previous study [18] robots were introduced with the grammatically neuter word “the system”, the general association of the grammatically masculine gendered word “the robot” might have influenced the results. Besides this possible general association of masculinity and robots in grammatically gendered languages, the question arises whether people perceive objects more gendered due to their grammatical gender [23]. Based on past research on the influence of grammatical gender on the perception of animate and inanimate entities [24, 25] it can be assumed that this might be also the case for robots. However, language effects on the participants side are currently a widely overlooked aspect in HRI. Both aspects, the appearance of the robot and language of participants could be an alternative explanation why robots were gendered more as male than female in the study by Roesler et al. [18].

With two online studies, we aimed to address the described methodological drawbacks of the earlier experiment [18], as well as further broaden the view to the connection of grammatical and perceived gender in HRI. Online studies were used as they offer the opportunity to effectively reach people from different countries, especially in times of the COVID-19 pandemic [26]. More precisely, we wanted to shed light on the role of native language as well as grammatical gender for the perception of robots’ gender. The first study, investigated to what extent anthropomorphism and native language might therefore have led to the male-robot bias [18]. Building on this, the second study examined whether the grammatical gender of words leads to an ascription of gender to robots. Moreover, we took a closer look whether this might be even reinforced by anthropomorphism. Accordingly, these studies aimed to clarify to what extent both factors language and anthropomorphism might contribute to a male-robot bias.

## Study 1

As the appearance of robots might have contributed to the previously found male-robot bias [18], we wanted to investigate if robots without obvious gender cues in their appearance, might still be perceived as male. In particular, we hypothesize that robots are perceived as more male gendered with a higher level of anthropomorphism. Further support for this hypothesis was provided by a recent study [27]. The results [27] showed that more anthropomorphic appearance leads to less perceived gender-neutrality of robots and that if robots are perceived gendered, then more often as masculine. Moreover, the results showed that masculinity is related to body manipulators like arms or legs and femininity to surface features like long hair or eyelashes. The study has been published after our study was conducted (see preregistration from May 2021 https://osf.io/xmv7s). In our opinion, the fact that two research groups have dedicated their research independently to the same research question further strengthens its relevance. Moreover, if the results point in the same direction, this would support the validity of the results by [27] and contribute to more reproducibility of HRI research [28].

In addition to the influence of visual robot features, also person-related aspects have to be considered as a potential impact factor on the perception of robots. In particular, evidence suggests that the language people speak influence the way they think about different entities. This is the core of the *linguistic relativity hypothesis* [29]. Regarding gender perceptions, many languages assign grammatical gender classes to nouns [24]. As stated already by other research on the topic of gender in HRI [15], this also applies to the word robot which is assigned a masculine grammatical gender in the German language. Even though the previous study used the grammatically neuter description “the system” [18], the mental linkage of the masculine word “the robot” might have been prevalent. It has already been shown that people form a mental representation of gender based on grammatical gender in German [30]. More precisely, German as a grammatically-gendered language and English as a natural gender language differ in their dependent forms. For example, articles, also match the gender of the noun in German, which is not the case in English [31]. However, while most nouns have no grammatical indication of gender, natural gender languages still distinguish gender through pronouns.

Moreover, in English, the mental representation of gender is still based on stereotypical associations even though the nouns describing the persons are not gendered (e.g., beauticians are stereotyped as female and police officers as male) [30]. Importantly, the stereotypical representation is representing a male-technology bias in English language, as for example computer scientists, technicians, and engineers are perceived as rather male than female [30]. This could also lead to a male-robot bias, even though the word has no grammatical gender. However, whether people speaking differently gendered languages, perceive robots as comparably male gendered, has not yet been researched. Therefore, results from German-speaking samples supporting the male-robot bias might be interpreted with caution in regard to generalizability to natural gender languages like English. Whereas occupational stereotypes exist in both languages [30], the German language is additionally grammatically gendered. Even though the robots were introduced with the neuter wording “the system” in the previous study [18] the association of the gendered word “the robot” might have influenced the results. Therefore, we hypothezised that robots are perceived more male gendered in a German sample compared to an English sample, even though the robots are introduced via neuter descriptions.

### Methods

#### Participants

A sample size of 80 with 40 participants per language condition was targeted to obtain .80 power to detect a small to medium effect size of .15 at the standard .05 alpha error probability. In total, 82 participants (53 women, 27 men, 2 non-binary individuals; mean age = 28.74, *SD* = 5.96) took part in the study. All of them provided usable scores, so none of the participants needed to be excluded.

Half of the participants were German-speaking. They were recruited through the university’s participant pool, which resulted in a mostly student sample as the pool is used by current and former students (28 women, 13 men, 0 non-binary individuals; mean age = 27.32, *SD* = 4.27). This half of the sample did not receive any monetary reimbursement, but could collect course credit.

The other half of the sample was recruited through the platform *Prolific* and received monetary reimbursement (£0.7). Only English monolinguals, i.e., people with English as their first and only language, took part (25 women, 14 men, 2 non-binary individuals; mean age = 30.17, *SD* = 7.04). This decision was made because there is some evidence that the gender system of other known languages with a higher representation of gender might influence the results in the direction of assigning more gendered characteristics [24].

As this effect is not assumed to exist vice versa from lower to higher representation of gender, and multilinguality is widespread in Germany, the German sample was not monolingual. Besides the language characteristics, the same inclusion criterion regarding age, ranging from 18 to 45, was used for both recruitment approaches.

#### Task and Materials

Participants’ task was to intuitively rate 15 different robotic systems in regard to their human-likeness and perceived gender. The term “the robotic system” was used in line with the earlier study [18]. This was done to not explicitly gender the stimulus material, as robotic system is grammatically neuter in both German and English. For stimulus material, a picture set of nine robots from an earlier experiment [18] was extended. The original stimulus material consisted of three different degrees of anthropomorphism (low vs. medium vs. high) each including three different robots. For the current study another six robots, two for each degree of anthropomorphism, were added to the set. This resulted in 15 robot pictures of which five were assigned to each degree of anthropomorphism.

Like the original set, pictures were derived from the Anthropomorphic roBOT (ABOT) database^1^ [32]. This database lists 251 robots that are currently on the market with ratings on how human-like they are perceived. Robots were chosen by this ABOT database’s Human-Likeness Score, ranging from 0 to 100, with 100 meaning the robot is perceived as completely human-like. Additionally, attempts were made to choose robots without obvious stereotypical gender markers in their design, such as hair cuts or make-up.

**Fig. 1:**
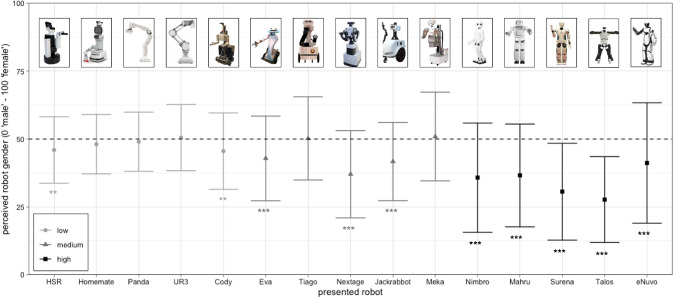
Mean and ± 1 SD of gender ratings for all robots. All robots in this study were colored in gray and white shades, and any brand names or logos were removed. Colors and shapes indicate degree of anthropomorphism: circle/light gray = low, triangle/medium gray = medium, square/black = high, and asterisks indicate significance of differences to the value 50 *p* values: *$p <.05$, **$p <.01$, ***$p <.001$

Looking at the new set of 15 robots, Human-Likeness Scores were comparable within each degree of anthropomorphism: All five low anthropomorphic robots’ scores ranged from 6 to 11. Medium anthropomorphic robots’ scores varied from 22 to 25 and high anthropomorphic robots’ from 47 to 51. Higher scores considerably above 50 were not included in the data set to avoid the *uncanny valley* [33], which states that robots with extensive human-like features are perceived as eerie. For an even closer resemblance, every robot picture was resized to 200x200 pixel, colored in gray and white shades and any brand names or logos were removed in *Adobe Photoshop*. The stimuli are depicted in Fig. 1.

#### Study Design

The study consisted of a 2 $\times$ 3 mixed design with the between-subjects factor language (German vs. English) and the within-subjects factor degree of anthropomorphism (low vs. medium vs. high).

#### Measures

A 5-item short version of the Individual Differences in Anthropomorphism Questionnaire (IDAQ) [4], which only included questions referring to technological devices, was used as a control variable. The items (e.g., “To what extent does the average robot have consciousness?”) were rated on a 0 *not at all* to 10 *very much* scale.

To measure perceived human-likeness as a manipulation check, the single item “How human-like do you perceive the appearance of this robotic system?” was used. Participants had to indicate the human-likeness of each robot on a scale ranging from 0 (*not at all human-like*) to 100 (*completely human-like*).

Perceived gender was measured on a continuum via the single item “How do you perceive the appearance of this robotic system?”. The scale to answer this question ranged from 0 (*male*) to 100 (*female*). This response format was used to not only gain a binary gender category [11], but to allow for more fine-grained degrees of perceived gender. Even though robots might only seldom be perceived as unambiguously male or female, they might be perceived male or female to a certain degree [27]. This item was used to combine both a forced direction of binary gender [11], as one anchor was male and the other one was female, and a fine-grained degree of each gender association [27].

#### Procedure

The online-study was conducted from May to June 2021 using *SoSciSurvey*. Participants rated a set of 15 robot pictures (five low, five medium, five high anthropomorphic robots) in regard to their perceived human-likeness and perceived gender in random order. The robots were presented via picture only without showing names of the brand or respective robot. The survey concluded with the short version of the IDAQ and demographic questions on age and gender. The entire procedure lasted approximately 5–10 min.

#### Statistical Analysis

First, the control variable was analyzed via two sample t-tests for a comparison between the English and German sample, as well as the comparison between female and male participants of each sample. The manipulation check was analyzed via a one-way repeated measures ANOVA with the within-subjects factor degree of anthropomorphism (low vs. medium vs. high). Subsequently, the dependent measure was analyzed via a two-way mixed ANOVAs with the between-subject factor language (German vs. English) and the within-subject factor degree of anthropomorphism (low vs. medium vs. high). Assumptions for the parametric tests were checked and some violations were found. However, as the F-test is relatively robust towards deviations from normal distribution and inhomogeneity of variances [34], particularly with equal group sizes [35] as was the case in the current study, the analyses were conducted as planned. In addition, we used the Greenhouse-Geisser correction to adjust for lack of sphericity in a repeated measures ANOVA.

### Results

#### Control Variable

The shortened 5-item version of the IDAQ showed an acceptable internal consistency for the original English version ($\alpha =.74$) and translated German version ($\alpha =.70$). To control for individual differences in the tendency to anthropomorphize between both samples an independent t-test was conducted. The analysis revealed no significant difference, (*t*(80) = $-$ 1.68, *p* =.098) between the mean IDAQ sum scores of the German (*M* = 9.41, *SD* = 4.72) and the English sample (*M* = 11.63, *SD* = 7.04). In addition, we checked for gender differences within each country. The English sample incorporated two non-binary individuals, which unfortunately needed to be excluded from the analysis due to the small group size. On a descriptive level, non-binary participants had a slightly lower tendency to anthropomorphize (*M* = 9.50, *SD* =6.36) compared to female (*M* = 11.92, *SD* = 6.54) and male (*M* = 11.43, *SD* = 8.33) participants. No significant difference was revealed between female and male participants in the English sample (*t*(37) = 0.20, *p* =.840). In line with this result, no significant difference was revealed between female (*M* = 9.96, *SD* = 4.81) and male (*M* = 8.23, *SD* = 4.48) participants in the German sample, (*t*(39) = 1.10, *p* =.280).

#### Manipulation Check

The analysis of human-likeness revealed a significant main effect of degree of anthropomorphism ($F{(1.74, 140.54)} = 565.92, p <.001, \eta ^2 = 0.686$). Bonferroni-adjusted post-hoc tests showed that the human-likeness of low anthropomorphic robots (*M* = 14.15, *SD* = 12.02) was perceived as significantly lower than of both, medium (*M* = 35.85, *SD* = 16.31, $p <.001$) and high anthropomorphic robots (*M* = 68.79, *SD* = 17.09, $p <.001$). Since the manipulation check was intended to verify the different degrees of anthropomorphism of the robots which were selected on the basis of the ABOT database [32], we conducted a correlation analysis with the score of the ABOT database. The mean perceived human-likeness (*M* = 39.60, *SD* = 23.76) was descriptively higher than the mean ABOT database score (*M* = 27.19, *SD* = 17.32), however, both scores correlated extremely high ($r = 0.98, p <.001$).

#### Dependent Variable

Results regarding the perceived gender showed a significant main effect of degree of anthropomorphism ($F{(1.95, 155.70)} = 61.20, p <.001, \eta ^2 = 0.251$). Bonferroni-adjusted post-hoc tests revealed that low anthropomorphic robots (*M* = 47.82, *SD* = 8.02) were perceived as significantly less male than medium (*M* = 44.54, *SD* = 9.03, $p =.032$) and high anthropomorphic robots (*M* = 34.37, *SD* = 12.62, $p <.001$). Moreover, medium anthropomorphic robots were perceived as significantly less male than high anthropomorphic robots ($p <.001$). Surprisingly, neither a significant main effect of language ($F{(1, 80)} = 3.11, p =.081, \eta ^2 = 0.021$) nor a significant interaction effect were found ($F{(1.95, 155.70)} = 0.72, p =.487, \eta ^2 = 0.004$).

#### Exploratory Analyses

To take possible interindividual differences into account, we conducted a correlation between the individual tendency to anthropomorphize and the manipulation check as well as the dependent variable. The analyses showed that the IDAQ sum score did neither correlate with the perceived human-likeness ($r = 0.04, p =.577$) nor with the perceived gender ($r = -0.08, p =.225$).

Besides the analysis of more or less gendered perceptions of different degrees of anthropomorphism, we extended the analysis on perceived gender to a more detailed level, independently for each single robot stimuli. Therefore we investigated, which robots were perceived as gender-neutral via Bonferroni corrected t-tests against a fixed value of 50 on the means of all 15 robots. The results are illustrated in Fig. 1. All of the highly anthropomorphic robot stimuli received mean ratings significantly smaller than 50 (all $p <.001$). Three of the medium anthropomorphic (all $p <.001$) and two of the low anthropomorphic robots (all $p <.005$) were rated significantly smaller than 50. Thus, only 5 out of 15 robots did not differ significantly from the gender-neutral rating of 50. Moreover, as can be seen in Fig. 1 all robots which differed significantly from 50 were perceived as male and not female.

It is noteworthy, that since conducting the study a paper was published that presents the humanoid ROBOts - Gender and Age Perception (ROBO-GAP) dataset [27]. The perceived robot gender was measured in this study with the mean values for femininity, masculinity and neutrality [27]. Taking together the values of the ROBO-GAP dataset and the current gender continuum score we performed correlation analyses. The analyses revealed no significant correlation of perceived robot gender with the mean femininity rating ($r= -0.12, p =.670$). However, a significant negative correlation was found for perceived robot gender and the mean masculinity rating ($r = -0.84, p <.001$), as well as a significant positive correlation for perceived robot gender and the mean neutrality rating ($r = 0.63, p =.010$).

### Discussion

The aim of the first online-study was to investigate whether robots are perceived differently gendered in samples with differently gendered languages and whether more anthropomorphism in robot design leads to a more gendered perception of robots.

The manipulation check revealed a successful manipulation of anthropomorphism. The overall descriptively higher ratings in this study compared to the ABOT database scores might be explained by the selected robots, as none of the robot stimuli had a ABOT database score higher than 51 [32]. The comparison between the robots might thus have set an anchor for the higher ratings. However, the high correlation between the measurement used in this study and the ABOT database score further strengthens the validity of our results.

The idea to use a shortened version of the IDAQ questionnaire was implemented as economic tool to detect possible group differences in the tendency to anthropomorphize [4] as this can influence the way people perceive technologies. The short-version showed an acceptable internal consistency and no group differences were revealed. Overall, the values were rather low around 10 on possible value from 0 to 50, which is not surprising as uniquely human attributes are transferred to non-living technological objects in the questions.

The analysis of language differences revealed that the German and English sample did not significantly differ in their perception of robots’ gender. This result strengthens the generalizability of research concerning gender in HRI conducted with German speaking participants beyond a German speaking population [18]. However, this statement can not be made about research which used a grammatically gendered reference to robots like [15]. In addition, the results fit the effect that a male-technology bias is present in both, grammatically-gendered language and natural gender language [30]. Nonetheless, the term robotic system, which was used in the study, might have masked an even stronger transfer of grammatical gender to robot gender in the German sample. The second study, therefore, addressed the question whether the explicitly grammatically gendered descriptions “the robot” in German might lead to an even more male gendered perception of robots than a neuter description (e.g., the robotic system).

The results of the first study consolidate the assumption that the found male-robot bias [18] is associated with robot appearance. As stated by recent research [27], robots are often perceived as masculine as long as they do not exhibit female interpreted surface features. The research of [27] resulted in the ROBO-GAP database as an extension of the ABOT database. The comparison of the current results with this new database leads to some promising insights.

First, robots that were rated neutral in our study are also classified as neutral in the ROBO-GAP dataset [27]. Second, all robots except for one, that were perceived as male in the current study were classified either as neutral or as masculine in the ROBO-GAP dataset. More precisely, all male perceived robots of the medium degree of anthropomorphism and one (i.e., Nimbro) of the high degree of anthropomorphism category were classified as neutral in the ROBO-GAP dataset. This difference could emerge from different methodological aspects like the number of robots participants rated (i.e., 15 vs. around 50), the maximum Human-Likeness Score (i.e., max. 51 vs. 97) [32], or the type of rating (i.e., one item with scale from 0 “male” to 100 “female” vs. three items for “feminine”, “masculine”, and “gender neutral” with 7-point Likert scales). Our exploratory analyses suggest that the type of rating, in particular, might be the reason for this difference. Whereas our measurement significantly correlated negatively with masculinity, and positively with neutrality, no significant correlation was found for femininity. By using a single item with opposing extrema, we might have suppressed ambiguities, which might have led to a more neutral rating in the ROBO-GAP dataset. Third and most interestingly, one robot (i.e., eNuvo) was perceived as male in our analysis and as feminine in the ROBO-GAP dataset. This can be explained by the fact that we colored all robots in grey and white shades, whereas [27] used the original color scheme. The original color can be described as metallic pink. Pink, besides blue, is one of the colors with the strongest color-gender linkage [36] and often clearly referred as “girls” color.

This finding further strengthens the assumption that anthropomorphic robot bodies are interpreted as male, as long as no surface features (e.g., hair, lips and color) are clearly contradicting this perception [27]. Moreover, this is a starting point for future research in regard to color in HRI. Whereas the whiteness of robots have been extensively criticized [37, 38], little is yet known about the social and ethical consequences of other color schemes in regard to the perception of robots. Until now, surface color was used to make the robot appearing to be more extroverted [12] or to better meet the users’ expectations [39]. However, it should be kept in mind that color might also elicit undesired gender stereotypes.

In conclusion, the first online-study investigated the relationship between anthropomorphic robot appearance and perceived gender. For anthropomorphic appearance, it can be stated that all robots with high degree of anthropomorphism and most robots with medium degree of anthropomorphism were perceived rather as male than neutral (or even female). This further strengthens the possible presence of a male-robot bias in HRI, as anthropomorphic body manipulators like legs or a torso lead rather to a perception of masculinity [27] than gender-neutral human-likeness. Importantly, this assumption is not limited to gendered languages (e.g., German), as no significant difference occurred in regard to perceived gender. However, the most puzzling question that remains open concerning language effects is whether this was related to the usage of the neuter wording “the system”, and whether using the explicitly masculine grammatically gendered wording would lead to different results. This research question was therefore targeted in the second study.

## Study 2

In contrast to English, many languages are grammatically gendered. Whereas some of them have masculine and feminine grammatical genders (e.g., Italian, Spanish, or Arabic), others like German also assign neuter ones. In grammatically gendered languages, the question arises whether the grammatical gender of inanimate objects (like robots) lead people to think of them in a gendered manner [23]. A variety of research in psychology, as well as linguistics, approached this question and investigated whether grammatical gender influences the representation of concepts [24]. The ascription of gender to inanimate objects were mostly investigated in comparison to the ascription of animal gender [25, 40]. For inanimate objects, the results are at least mixed, as studies with inanimate targets showed only partially support for the assumption that grammatical gender influences the gender association [24]. Most studies used words of household items (e.g., spoon, razor, pencil) or tools (e.g., saw, screwdriver, ladder) for the inanimate category [23, 25, 40]. Even though these object might be anthropomorphized under some circumstances [41] the effect is more known to be applied to other humans, animals, and in particular to non-living objects like robots [4].

In contrast to objects used in these earlier studies (e.g., spoons), the observation of and interaction with robots activates social brain areas [42]. This clearly contrasts them from other non-living objects like household items. Moreover, this leads to the assumption that robots might trigger an association of ascribed gender comparable to living entities like animals. This association might be even more pronounced for more anthropomorphic robots, as one of the important aspects for artificial entities to appear social is human-like appearance [42]. As the first study and other research [27, 43] vividly illustrated, anthropomorphism often goes along with a more (male) gendered appearance. However, empirical evidence for the attribution of gender to gender-neutral robots is rather scarce.

So far, no study has investigated the effect of grammatical gender and anthropomorphism on perceived robot gender. The second study therefore aims to fill this research gap. In addition to the gender measurement of the first study, we wanted to incorporate a more subtle and less biased approach to investigate whether gender is attributed to robots [43]. In line with earlier research, [18] we used a naming technique and asked participants to name the respective robot. This open format enabled participants to give any name they could imagine, like neutral or technical ones. Based on the findings on grammatical gender and linguistic relativity [24], we assumed that robots introduced via a grammatical gender (masculine/ feminine) are perceived as more gendered in the respective direction. We hypothesized that robots introduced via a masculine grammatical gender are perceived as more male than robots introduced via a feminine or neuter grammatical gender. Vice versa, robots introduced via a feminine grammatical gender are perceived as more female than robots introduced via a masculine or neuter grammatical gender. Moreover, we hypothesized that more gendered names (female/male) are assigned to robots introduced by the respective grammatical gender. Lastly, we hypothesized that all differences are more pronounced for robots with higher degrees of anthropomorphism compared to robots with lower degrees of anthropomorphism.

### Methods

#### Participants

A sample size of 111 German participants (37 per grammatical gender condition) was targeted to obtain .80 power to detect a small to medium effect size of .15 at the standard .05 alpha error probability. In total, 134 participants took part in this study. Applying an attention check resulted in the exclusion of 23 participants. This resulted in the targeted sample size of 111 participants (44 women, 65 men, 2 non-binary individuals; mean age = 32.79, *SD* = 10.83) which were equally distributed between the three groups. The sample was recruited through the platform *Prolific* and participants received monetary reimbursement (£0.85).

#### Task and Materials

The introduction of the robot was kept nearly the same and only the relevant wording was changed to the word with the respective grammatical gender. We used the *the robotic machine* as feminine, *the robotic automate* as masculine and *the robotic system* as neuter reference of the robot. The respective wording was applied throughout the whole study (introduction, instructions for the tasks and gender perception items).

The naming task was to intuitively name four different robots. The four images of real world robots, i.e., *Panda, UR3* representing the low anthropomorphic category and *Tiago, Meka M1*, representing the medium anthropomorphic category, were presented to each participant. The pictures used in this study were the same as in study one. Those images were chosen based on the first study and the ROBO-GAP database [27], as they are all perceived as gender-neutral. The highly anthropomorphic category was excluded as all robots of this category were perceived as rather male and not gender-neutral in the first study. Besides the naming task, participants needed to rate the perceived gender of the four robots.

#### Study Design

The study consisted of a 3 $\times$ 2 mixed design with the between-subjects factor grammatical gender (feminine vs. masculine vs. neuter) and the within-subjects factor degree of anthropomorphism (low vs. medium).

#### Measures

An attention check question was included in order to check whether participants had correctly understood the specific definition of their condition. Participants had to answer the question: “What was presented to you on the pages of this questionnaire?” via a forced choice between the three options *the robotic machine*, *the robotic system*, and *the robotic automate*. The order of the response options was random.

As control variable, the same 5-item short version of the Individual Differences in Anthropomorphism Questionnaire (IDAQ) [4], as in study one was used.

As dependent variable, again, perceived gender was measured on a continuum via the single item used in study one on a scale from *0 to 100* to investigate whether the respective grammatical gender leads to a shift towards a more male/female perception of robots. In addition, a naming technique was used to measure assigned gender. Given names were coded into four categories by three raters working in our research group. The categories were *female, male, functional* names, and *nicknames*. As an assessment of the inter-rater reliability of the three raters, *Fleiss’ Kappa* was computed. After the first iteration, inter-rater reliability was substantial for names given to *Panda* (*K*$_{Fleiss}$ =.766), *UR3* (*K*$_{Fleiss}$ =.761), *Tiago* (*K*$_{Fleiss}$ =.792), and *Meka M1* (*K*$_{Fleiss}$ =.799). In case of discordance, the rating two out of three raters agreed upon was applied as the final rating. From these categories, frequencies of given names were computed for each condition.

#### Procedure

The 5-min online study was carried out using the platform *SoSciSurvey* in October 2022. After consenting to the study, participants were presented with the group specific word framing and the instructions. First, participants named the set of four robot pictures (two low and two medium anthropomorphic robots). Each robot was presented on a separate page and the order of the pictures was random. Afterwards, they rated the perceived gender of the four robots, which were again presented in random order. The survey concluded with the attention check, a short version of the IDAQ, and demographic questions on age and gender. The entire procedure lasted approximately 5 min.

### Results

#### Control Variable

The German IDAQ short version showed a good internal consistency ($\alpha =.80$). No significant differences were revealed between the experimental groups, as the results of a between-subjects ANOVA revealed ($F{(2, 108)} = 1.58, p =.211, \eta ^2 = 0.028$). In addition, we checked for gender differences of the sample in regard to the IDAQ. The sample incorporated two non-binary individuals, which unfortunately needed to be excluded from the analysis due to the small group size. On a descriptive level, non-binary participants had a higher tendency to anthropomorphize (*M* = 13.00, *SD* = 2.83) compared to female (*M* = 7.20, *SD* = 3.30) and male participants (*M* = 8.52, *SD* = 6.24). A two sample t-test showed no significant differences between female and male participants (*t*(107) = $-$ 1.28, *p* = .202).

#### Dependent Variables

The perceived gender on the continuum item was analyzed via a 3x2 mixed ANOVA with the between-subjects factor grammatical gender (feminine vs. masculine vs. neuter) and the within-subjects factor anthropomorphism (low vs. medium). The analysis revealed no significant main effect of grammatical gender (*F*(2, 108) = 0.20, $p=.822, \eta ^2 = 0.002$). The robots in the grammatically feminine (*M* = 42.20, *SD* =17.17), masculine (*M* = 43.67, *SD* = 16.23), and neuter (*M* = 43.63, *SD* = 16.84) condition were descriptively perceived as comparably (male) gendered. In addition neither the main effect of anthropomorphism ($F{(1, 108)} = 0.15, p =.699, \eta ^2 = 0.001$), nor the interaction effect were significant ($F{(2, 108)} = 2.38, p= .097, \eta ^2 = 0.023$).

Overall, the naming of the robots showed a preference for functional names (49.10%) like “supporter”, “power arm”, or “helper”, as well as nicknames (29.28%) like “monday”, “tilly”, or “fiffy” over male (17.34%) and female names (4.28%). Moreover, participants assigned more male names such as “Mike”, “Tim”, or “Rupert” than female names like “Lucy”, “Sue”, or “Stella”. All reported percentages refer to the percentage of the respective name of all assigned names in total. The main effect of grammatical gender as well as the interaction effect of grammatical gender and anthropomorphism were analyzed via Chi-squared tests. If significant differences were revealed, Bonferroni corrected post hoc pairwise comparisons were conducted to compare the respective conditions of the three level factor as well as combinations of factors for the hypothesized interaction.

Female names were not assigned significantly ($\chi ^2(2)$ = 1.37, *p* =.505) more often in the feminine grammatically gender condition (0.90%) compared to both the masculine (1.58%) and neuter (1.80%) condition. In addition, no significant differences were revealed for the 3 (grammatical gender) $\times$ 2 (anthropomorphism) table of female names ($\chi ^2(2)$ = 0.09, *p* =.956).

The analysis of assigned male names just failed to reach the conventional level of significance ($\chi ^2(2)$ = 5.79, *p* =.055). On an descriptive level male names were assigned more often in the masculine (7.88%) grammatical gender condition compared to the feminine (5.41%) gender and especially neuter (4.05%) gender condition. No significant effect was revealed for the assignment of male names in differently grammatically gendered conditions, depending on the anthropomorphism of the robot ($\chi ^2(2)$ = 0.21, *p* =.901).

#### Exploratory Analyses

To investigate the name options apart from binary gendered ones, we analyzed the assignment of nicknames and functional names via the same procedure as the male and female names.

The analysis of assigned nicknames revealed significant differences between the grammatical gender conditions, ($\chi ^2(2)$ = 6.57, *p* =.037). Post hoc tests revealed that significantly more nicknames were assigned in the masculine (11.94%) grammatical gender condition compared to the neuter (6.76%, *p* =.035) condition. No further significant differences were revealed in regard to the comparison of the feminine grammatical gender condition, and both other conditions (10.58%, both $ps >.157$). Furthermore, adding the factor anthropomorphism did not result in significant differences ($\chi ^2(2)$ = 0.97, *p* =.615).

For functional names the analysis showed significant differences between the grammatical gender conditions, ($\chi ^2(2)$ = 10.47, *p* =.005). In particular, significantly more functional names were ascribed in the neuter (20.72%) grammatical gender condition compared to the masculine gender one (11.94%, *p* =.004). Moreover, the ascription of functional names did not significantly differ between the feminine and other grammatical gender condition (16.44%, both $ps >.225$).

### Discussion

The objective of the second study was to examine the role of grammatical gender and anthropomorphism for the perception of robot gender. Based on previous research on other non-human entities (e.g., animals) [24] which trigger mind perception [44], we supposed that grammatical gender influences the gender perception of robots.

The analysis of the gender continuum score did not support this assumption, as no significant differences in perceived gender were revealed for robots introduced via feminine, masculine or neuter wordings. This is only partially supported by the analysis of assigned names, as no significant differences between the frequency of assigned female names were found for differently grammatically gendered robots. However, the analysis of the frequency of assigned male names revealed an interesting trend. Participants tended to assign more male names, and significantly more often assigned nicknames if robots were introduced grammatically masculine gendered compared neuter gendered. In turn, significantly less functional names were assigned in the masculine condition compared to the neuter one.

These results at least partially support the hypothesis that the language used to reference objects influences the way people think about these objects [24, 29]. In regard to this hypothesis two points in particular should be emphasized. First, the influence of grammatical gender was only revealed in the naming technique but not in the gender continuum score. This again shows that measuring perceived gender via questionnaires is having its shortcomings [43]. Especially for subtle gender cues like the grammatical gender a more subtle measurement like the naming technique seems to be more suitable [18]. Second, only the masculine compared to the neuter grammatical gender introduction lead to differences. The lack of differences of the assignment of female names may mainly be due to the fact that almost no female names are assigned. The result that under 5% of assigned names were female is in line with earlier research using the naming technique for robots without obviously female appearance cues.

So generally, the results illustrate that if gendered names are assigned than male ones. This pattern of results is consistent with the previous literature [18]. The results provide supporting evidence that the strength of the male-robot bias might depend on grammatical genderedness. Whereas a masculine reference seems to increase the male-robot bias, a neuter one seems to be a opportunity to decrease this bias and foster more functional attributions. These results suggest that using neuter grammatically-gendered descriptions of robots is a promising practical approach to reduce the male-robot bias.

Contrary to the assumptions, anthropomorphism did not play a meaningful role for the perceived gender. However, this result needs to be treated with caution as the stimuli only incorporated low and medium anthropomorphic robots [32]. The aspect that there are differences in the effectiveness of grammatical gender between animate and inanimate objects [24], encourages further studies on the role of anthropomorphism. Future studies should therefore investigate if higher degrees of anthropomorphism of gender-neutral robots in combination with a grammatically male gendered ascription lead to a even more pronounced male-robot bias. Moreover, the connection of gendered appearance [27] and grammatical gender opens up multiple avenues for future research.

## General Discussion

Taken together, the findings of the two online studies indicate factors that can reinforce a male-robot bias [18]. The results together with other recent research [27] strongly imply that anthropomorphic appearance, without obvious gender cues is associated with the perception of masculinity. Perhaps, this effect is not limited to gendered languages only (e.g., German). Furthermore, the results indicate that the male-robot bias is increased by an introduction of robots in a grammatically gendered way. This is especially problematic in languages where the word “the robot” is masculine (e.g., German or Italian). Even though a mismatch of robot gender and occupational stereotype might be beneficial in some contexts [15], a gender-neutral appearance and neuter grammatical reference offer the opportunity to reduce gender stereotypes in HRI.

Although the present results clearly support the existence of a male-robot bias, as well as possible moderating factors, it is appropriate to recognize several potential limitations. A major limitation concerns the type of exposure [1]. The two-dimensional depictions of robots used in this study, cannot represent important aspects anthropomorphism in HRI like communication, movement, or task context [1]. Moreover, depictions can also skew the perception of the robot gender as body aspects as height can hardly be represented. Another limitation is that the gender continuum scale was used as only gender assessment in the first study. The results of the second study showed the importance to incorporating more subtle gender measurements [43] like the naming technique [18]. Besides issues of measuring robot gender, the analysis of participants’ gender also presents us with challenges, which should be addressed in more research. Even though it is an advantage that gender non-conforming participants took part in the studies [43], the group size in both studies was to small to include these participants in the analysis of the control variable. At least, in all other analyses gender non-conforming participants were included.

Despite these methodological pitfalls, the present studies have enhanced our understanding of the male-robot bias and contributes to a growing body of evidence suggesting that even robots without obvious gender cues can elicit a perception of masculinity. To reduce this bias, robot design and language aspects should consciously be implemented gender-neutral.

## Data Availability

The preregistration, ethical evaluation, stimuli and collected data can be accessed via https://osf.io/dnb3z/ for study one and via https://osf.io/7dy68/ for study two, to ensure transparent and reproducible research.

## Footnotes

1 http://abotdatabase.info/collection, version from 03/2019.
